# ATP2B1-AS1 Promotes Cerebral Ischemia/Reperfusion Injury Through Regulating the miR-330-5p/TLR4-MyD88-NF-κB Signaling Pathway

**DOI:** 10.3389/fcell.2021.720468

**Published:** 2021-10-12

**Authors:** Lei Wang, Ying Tan, Ziyu Zhu, Jun Chen, Qiang Sun, Zhibin Ai, Chunqi Ai, Yu Xing, Guohou He, Yong Liu

**Affiliations:** ^1^Department of Human Anatomy, Histology and Embryology, Institute of Neurobiology, Health Science Center, Xian Jiaotong University, Xi’an, China; ^2^Department of Neurology, Affiliated Taihe Hospital of Hubei University of Medicine, Shiyan, China; ^3^Department of Neurology, Affiliated Taihe Hospital of Xian Jiaotong University Health Science Center, Shiyan, China; ^4^Department of Laboratory Medicine, Affiliated Taihe Hospital of Hubei University of Medicine, Shiyan, China; ^5^Department of Mental Health Centre, Affiliated Taihe Hospital of Hubei University of Medicine, Shiyan, China; ^6^Department of Medical Image Center, Taihe Hospital, Hubei University of Medicine, Shiyan, China

**Keywords:** ATP2B1-AS1, cerebral ischemia/reperfusion, miR-330-5p, long non-coding RNA, TLR4, inflammation

## Abstract

We aim to explore the expression and function of long non-coding RNA (lncRNA) ATP2B1-AS1 in a cerebral ischemia/reperfusion (I/R) injury. In this study, we established a middle cerebral artery occlusion/reperfusion (MCAO/IR) rat model and an OGD/R PC12 cell model to evaluate the expression and role of ATP2B1-AS1 in the cerebral I/R injury. We found that the expression of ATP2B1-AS1 was upregulated in both *in vitro* and *in vivo* cerebral I/R injury models. Knockdown of ATP2B1-AS1 increased the cell viability, inhibited apoptosis, and decreased the expressions of inflammation cytokines. The target of ATP2B1-AS1 was predicted and validated to be miR-330-5p. MiR-330-5p abrogated the regulatory effect of ATP2B1-AS1 on cell viability, apoptosis, and cytokines of OGD/R PC12 cells. Furthermore, the results showed that miR-330-5p targeted TLR4, which was also upregulated in the infarcted area of MCAO/IR rats and OGD/R PC12 cells. Overexpression of ATP2B1-AS1 increased the expressions of TLR4, MyD88, and NF-κB p65 of OGD/R PC12 cells, while the effect of ATP2B1-AS1 was abrogated by miR-330-5p. In addition, knockdown of ATP2B1-AS1 decreased the latency time, increased the time of passing the platform position, reduced the cerebral infarct volume, decreased neurological deficit scores, and reduced the number of damaged neurons of MCAO/IR rats that were subjected to the Morris water maze test. Taken together, our study indicates that ATP2B1-AS1 may be an attractive therapeutic target for the treatment of cerebral ischemic injuries.

## Introduction

Ischemic cerebral vascular disease can cause a different degree of neurological dysfunction and is considered to be a serious threat to public health for its high incidence, high morbidity, high disability, and high mortality characteristics (Zhong et al., 2019; Cai et al., 2021). Cerebral ischemia-reperfusion (I/R) injury refers to the phenomenon that the cerebrum suffers from a permanent or transient ischemia and then suddenly restores the blood supply (Block et al., 2020). The function of the cerebrum not only failed to recover, but more serious neurons dysfunction occurred. Therefore, how to reduce cerebral I/R injury is a key problem that urgently needs to be solved in the treatment of ischemic cerebral vascular disease. Cerebral I/R injury involves the participation of various pathophysiological processes, such as oxidative stress, calcium overload, glutamate-mediated excitotoxic damage, inflammatory reaction, etc. (Naderi et al., 2020). Increasing evidence has shown that the overexpression of inflammatory factors and infiltration of inflammatory cells are observed after cerebral I/R, triggering a series of inflammatory reactions (Wu L. et al., 2020). Studies have revealed that some substances that are released from the damaged cells, blood vessels, and extracellular matrices during cerebral ischemia would activate the Toll-like receptor 4 (TLR4)-mediated downstream signaling pathway (Rahimifard et al., 2017). TLR4 is an inflammatory signaling receptor, and its mediated signaling pathways, such as the TLR4/MyD88/NF-κB signaling pathway, play an important role in the inflammatory cascade in various I/R-induced vital organs injuries (Li J. et al., 2019).

Recently, increasing evidence suggests that non-coding RNAs that are once considered to have no biological function, such as microRNAs (miRNAs) and long non-coding RNAs (lncRNAs), are functionally active as physiological and pathological regulation molecules (Vasudeva et al., 2021). Numerous studies have shown that the lncRNAs participate in many important regulatory processes, such as epigenetic, transcriptional, and post-transcriptional regulation. LncRNA can interfere with the miRNA pathway and function as a natural miRNA sponge to reduce the binding of endogenous miRNA and its target genes (Vasudeva et al., 2021). A lot of scientists are focused on the biological functions of lncRNAs in the tumorigenesis, metastasis, and progression of various tumors (Fattahi et al., 2020). Several reports about the role of lncRNAs in a cerebral I/R injury were also published. For example, Xu et al. (2016) has identified a lncRNA, CAMK2D-associated transcript 1 (C2dat1), that regulated the expression of CaMKIIδ and promoted neuronal survival following cerebral ischemia. Song et al. (2019) reported that lncRNA H19 induced cerebral I/R injury via the activation of autophagy involving the DUSP5-ERK1/2 axis. Yin et al. (2019) reported that lncRNA SNHG12 attenuated cerebral I/R injury by targeting a miRNA, miR-199a, to upregulate SIRT1 and activate the AMPK signaling pathway.

ATP2B1-AS1, also named LINC00936, is a 3,626 bp long lncRNA, which is located in chr12:89,708,954–89,713,726 (Song et al., 2020). It has been investigated that ATP2B1-AS1 was highly expressed in mice with acute myocardial infarction, and the suppression of ATP2B1-AS1 presented a protective effect against a myocardial infarction injury in an *in vitro* model (Song et al., 2020). Sheng et al., 2020) reported that ATP2B1-AS1 was predicted to interact with TLR2 and participate in the Toll-like receptor signaling pathway, and regulated acute myocardial infarction. ATP2B1-AS1 has also been found to be a crucial regulator in mediating renal interstitial fibrosis and oxidative stress in chronic renal failure (Chen et al., 2018). To our knowledge, there are few reports on the expression and biological function of ATP2B1-AS1 in cerebral I/R injury. In this study, we first investigated the expression and biological function of ATP2B1-AS1 in a cerebral I/R injury and its potential therapeutic value with an MCAO/IR rat model and an oxygen and glucose deprivation-reoxygenation (OGD/R) PC12 cell model. OGD/R-treated neuron is a common model *in vitro* to imitate a MCAO/R-induced cell injury *in vivo* (Gong et al., 2017). We highlighted that ATP2B1-AS1 promoted cerebral I/R injury as a competing endogenous RNA for sponging miR-330-5p to activate the TLR4-MyD88-NF-κB signaling pathway. Suppression of ATP2B1-AS1 expression showed a neuroprotective effect.

## Materials and Methods

### Cell Lines and Animals

The PC12 cell was purchased from CCTC (MD, United States) and cultured in DMEM containing 10% FBS and 1% antibiotics at 5% CO_2_ at 37°C. Male Sprague-Dawley rats (SD, 220 ± 10 g, 8–10 weeks) were obtained from Shanghai Laboratory Animal Center (Shanghai, China). The rats were housed in a standard laboratory condition (temperature 22 ± 2°C and humidity 50–60%). All animal experiments and procedures were performed in accordance with the guidelines in the Histology and Embryology, and Institute of Neurobiology, Xian Jiaotong University Health Science Center. Institutional Animal Use Policy and approved by the Institutional Animal Care and Use Committee of Histology and Embryology, and Institute of Neurobiology, Xi’an Jiaotong University Health Science Center.

### Middle Cerebral Artery Occlusion/Reperfusion and Animal Treatment

The SD rats were subjected to MCAO/IR operations according to the previous description (Simion et al., 2019). Briefly, the rats were randomly divided into Sham, MCAO/IR (reperfusion for 12, 24, and 48 h), MCAO/IR + shNC, and MCAO/IR + sh-ATP2B1-AS1 (*n* = 6). The rats were anesthetized with isoflurane (3% initially, 1–1.5% maintenance) in N_2_O and O_2_ (3:1) and fixed on a thermostated hot plate. Then, the neck was shaved and performed a midline incision, the fascial muscles were separated, and the common carotid artery (CCA), internal carotid artery (ICA), and external carotid artery (ECA) were exposed. A nylon suture with a heat-blunted end was inserted into the ICA from the CCA and then forwarded to the origin of the ECA until it occluded the origin of the middle cerebral artery (MCA). Consequently, the blood flow to the right MCA was blocked. After occlusion for 1 h, the suture was carefully removed from the vessel for reperfusion for 12, 24, and 48 h (if not specified, the default reperfusion time is 24 h). For the sham group rats, the right MCA was separated but without a suture insertion. Then, the rats were anesthetized and brains were removed immediately or restored and subject to for further study.

### *In vivo* ATP2B1-AS1 Knockdown

The lentiviral vectors containing shRNA against ATP2B1-AS1 (shATP2B1-AS1: 5′-GCCATTACGGCTCAATGCA-3′) non-targeting control (shNC: 5′-CGGATATCGGCACTAACTCT-3′) were designed and synthesized by GenePharma (Shanghai, China). For the cell experiment, the transfection was performed by the Lipofectamine^TM^ 2000 kit according to the instructions of the manufacturer. For the rat experiment, the injection of shATP2B1-AS1 and shNC was going through the MCAO surgical procedures. Briefly, lentiviral particles containing shATP2B1-AS1 (1 × 10^8^ TU/mL, Dharmacon) or shNC were mixed with the Entranster^TM^ *in vivo* solution (0.5 μg/μl), and then administrated by tail venous injection at the beginning of reperfusion.

### Neurological Scoring

The neurological function was evaluated using a Zea Longa (Chai et al., 2020) 5-scoring system for 3–7 d after the MCAO surgical procedure, as follows: no neurological defect symptom was scored 0, cannot fully extend left front paw was scored 1, rotated to the left during walking was scored 2, tilted to the left during walking and stood unstably was scored 3 (Block et al., 2020), and no self-awareness and cannot be self-issued was scored 4 (Naderi et al., 2020). A single observer blinded to the group assignment performed the neurological testing.

### Morris Water Maze Behavioral Assessment

The Morris water maze behavioral assessment was performed according to the previous description (Zhao et al., 2020). The Morris water maze is a circular pool with a diameter of 130 cm and a height of 50 cm, filled with water to a depth of 30 cm. The water maze was divided into four equal quadrants (I–IV). In the fourth quadrant, a plexiglass platform with a diameter of 11 cm and a height of 29 cm was placed. The water maze was covered with blue curtains, and visual cues were deposited at various locations around the maze. A camera attached to the computer system is placed above the labyrinth to record the trajectory of the rats simultaneously. For the positioning test, all rats were trained in the water maze for 4 days. Firstly, a quadrant was randomly selected as the water inlet point and the rats were slowly placed in the water facing the pool wall, then the time (latency period) and swimming trajectory of the rats to find the plexiglass platform was recorded. If the rat did not find the platform within 60 s, then, the rat was guided to the platform with a glass rod and the latency period was recorded as 60 s. After 30 s of rest, the next quadrant test was continued. The water temperature was maintained at 23 ± 3°C during the test. For the space exploration test, on the fifth day, the platform was removed, and the rats were placed in the quadrant of the platform in the water, and the time of the rats staying in the fourth quadrant within 60 s and the number of crossing the platform were recorded.

### 2,3,5-Triphenyltetrazolium Chloride Staining Assay

The infarct volume of the brain was determined by TTC (Sigma, MO, United States) staining. In brief, at the end of the experiment, the rats were sacrificed after being anesthetized, the brains were rapidly removed and frozen at –20°C for 1 min and then sectioned into six pieces (2 mm/piece) along the coronal plane. The sections were stained with TTC (2%) for 30 min at 37°C protected from light, then TTC was removed and 4% paraformaldehyde was added to fix for 24 h. The normal brain tissues were stained red while the infarcted tissue remained unstained (white). The infarct volume was measured using AlphaEase.

### Nissl Staining

The removed brain was fixed with 4% paraformaldehyde for 24 h, then dehydrated, transparent, embedded in paraffin, sectioned, and stained with 1% cresyl violet. The images were captured by a light microscope.

### PC12 Cell Treatment and Transfection

An OGD/R model on the PC12 cell was used to mimic ischemic-like conditions *in vitro*. MiR-330-5p mimics for the overexpression of the miR-330-5p level or mimic control (miR-NC), ATP2B1-AS1 overexpression pcDNA3.1 vector (ATP2B1-AS1) or its control pcDNA3.1 vector (NC), as well as ATP2B1-AS1 knockdown vectors (shRNA#1, shRNA#2, shRNA#3) or its control vector (shNC) were designed and synthesized by GenePharma (Shanghai, China). The synthesized sequence was cloned into the plasmid vector pcDNA3.1. Briefly, the cells or the stably transfected cells with the specific vectors were cultured with a serum/glucose-free DMEM medium in a temperature-controlled (37°C) anaerobic chamber. After OGD exposure, the medium was replaced with normal DMEM containing glucose and FBS under a normoxic condition for 12, 24, and 48 h to reoxygenation. The LDH level and caspase-3 activity in the supernatant was determined by a commercial assay kit according to the instruction of the manufacturer.

### ELISA Assay

Part brain tissues were homogenized ultrasonically on ice for 30 min, centrifuged at 11,000 g for 15 min (4°C), and the supernatant was collected. The content of TNF-α, IL-1β, and IL-6 in the supernatant was determined by the ELISA kit following the instruction of the manufacturer.

### Flow Cytometry Assay

The apoptosis of the cells was determined by the calcein-AM/PI double stain kit (Dojindo, Tokyo, Japan). Briefly, the cells were collected after the specific process and washed with the assay buffer twice. The cells were adjusted to a suspension at 1 × 10^5^–1 × 10^6^ cells/ml using the assay buffer. Then, the calcein-AM/PI working solution was added and incubated for 15 min at 37°C. The apoptosis rate was determined by flow cytometry.

### Target Prediction and Dual-Luciferase Reporter Assay

The potential target of ATP2B1-AS1 was predicted by StarBase and the potential target of miR-330-5p was predicted by Targetscan. The binding was verified by the dual-luciferase reporter assay. HEK-293T cells were co-transfected with wild-type or mutant of ATP2B1-AS1 or TLR4 and miR-330-5p mimics (miR-NC mimics as a control) or not, respectively. Luciferase activity was determined on an illuminometer (Berthold, Germany).

### RNA Immunoprecipitation Assay

The binding relationship between endogenous ATP2B1-AS1 and miR-330-5p in the PC-12 cells was detected by a RIP RNA-binding protein immunoprecipitation kit (Millipore, MA, United States). Briefly, the cells were collected after washing with cold PBS and the RIP lysis buffer was added. Then, the suspension was centrifuged and 100 μl cell lysates were transferred to the RIP immunoprecipitation buffer which contained Ago2-conjugated magnetic beads, the mouse IgG as a negative control (Millipore, MA, United States). The magnetic beads washed with the RIP wash buffer and then incubated with proteinase K at 55°C for 30 min. The RNA was extracted for qRT-PCR analysis.

### qRT-PCR

The total RNA from the cells or tissue samples was extracted with a TRIzol reagent extraction kit (Invitrogen, Karlsruhe, Germany). The reverse transcription was performed with the SuperScript III First-Strand Synthesis system (Invitrogen, Karlsruhe, Germany). Quantitative assay of gene expressions was performed by an SYBR Green qPCR Kit (Finnzymes, Espoo, Finland) and an ABI 7500 real-time PCR system (Applied Biosystems, Foster City, CA). The gene expressions were normalized to the β-actin and calculated using the 2^–ΔΔCT^ method. The specific primer sequences were designed and synthesized by GenePharma. The primers for ATP2B1-AS1, miR-330-5p, TLR4, GAPDH, and U6 were listed as follows: ATP2B1-AS1 (Forward, 5′-GCTCTGACGTCTGTGTTTCCA-3′; Reverse, 5′ -AAGTGAAGGGCGTCCCACT-3′); miR-330-5p (Forward, 5′ -GTCTCTGGGCCTGTGTC-3′; Reverse, 5′-TCCTCCTCTTCC TCTCCATT-3′); TLR4 (Forward, 5′-GCAGTTTCTGAGCAG TCGT-3′; Reverse, 5′-CCTCCCACTCCAGGTAAGT-3′); GAPD H, (Forward, 5′-AGGTCGGTGTGAACGGATTTG-3′; Reverse, 5′-GGGGTCGTTGATGGCAACA-3′); U6, (Forward, 5′-ACCC TGAGAAATACCCTCACAT-3′; Reverse, 5′-GACGACTGAGC CCCTGATG-3′).

### Western Blot

The cell or tissue sample was lysed with an RIPA lysis buffer containing 1 mmol/L PMSF. Then, the homogenates were prepared and centrifuged at 12,000 rpm for 10 min at 4°C. The protein concentration was determined by the BCA method. Non-specific binding was blocked with 5% skimmed milk for 1.5 h, and the membranes were incubated with diluted primary antibodies against Caspase-3 (ab32042, 1:800; Abcam, Cambridge, MA, United States), TLR4 (ab13556, 1:800; Abcam), MyD88 (ab133739,1:800; Abcam), NF-κB p65 (ab207297, 1:800; Abcam), and β-actin (ab8226, 1:1,000; Abcam) overnight at 4°C and in the secondary antibody for 1 h at room temperature. The signals were determined by the Amersham prime ECL Plus detection system (Pittsburgh, PA).

### Statistical Analysis

All data were presented as means ± SD. Statistical analysis was conducted by one-way ANOVA followed by Tukey’s *post hoc* test with SPSS 13.0. Statistical significance was accepted at *P*-values < 0.05.

## Results

### ATP2B1-AS1 Is Upregulated in Middle Cerebral Artery Occlusion/Reperfusion Rats and Oxygen and Glucose Deprivation-Reoxygenation PC12 Cells

**FIGURE 1:**
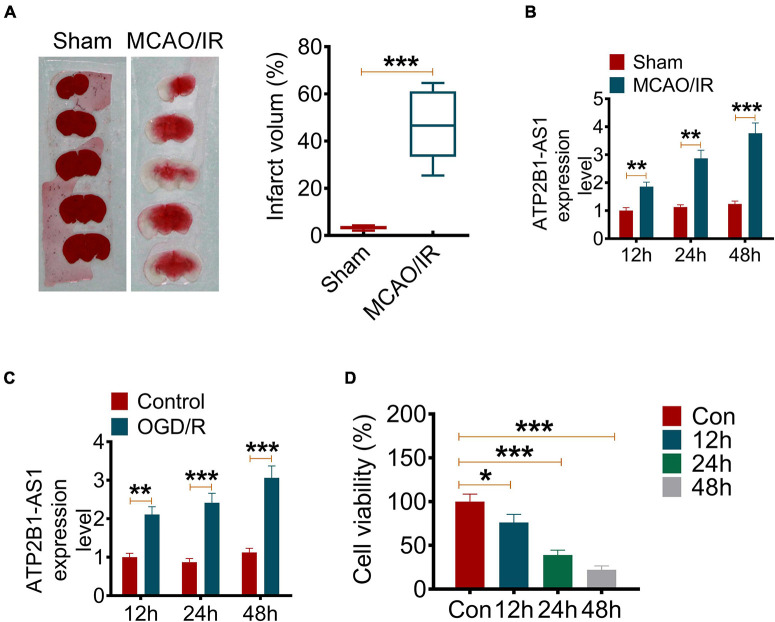
The ATP2B1-AS1 expression in MCAO/IR rats and OGD/R PC12 cells. **(A)** The cerebral infarct volume was detected by TTC staining. The representative images of brain slices of the Sham and MCAO/IR group rats are present on the left and the quantification of infarct volume on the right. **(B)** The mRNA expression of ATP2B1-AS1 in MCAO/IR rats for reperfusion for 12, 24, and 48 h was determined by qRT-PCR. **(C)** The mRNA expression of ATP2B1-AS1 in OGD/R PC12 cells for reoxygenation for 12, 24, and 48 h was determined by qRT-PCR. **(D)** The cell viability of OGD/R PC12 cells for reoxygenation for 12, 24, and 48 h was determined by the MTT method. *P < 0.05, **P < 0.01, ***P < 0.001.

In the present study, we established an MCAO/IR rat model to evaluate the expression and role of ATP2B1-AS1 in a cerebral I/R injury. The infarct volume of the brain of MCAO/IR rats was determined by TTC staining. As shown in Figure 1A, MCAO/IR rats showed a larger cerebral infarct volume compared with the Sham group rats significantly (*P* < 0.001). The ATP2B1-AS1 expression in MCAO/IR rats for different reperfusion times (12, 24, and 48 h) was also investigated. As shown in Figure 1B, it showed a similar expression level of ATP2B1-AS1 for different reperfusion times in the Sham group rats, while the expression level of ATP2B1-AS1 was increased significantly in the MCAO/IR group rats and increased in a time-dependent manner for reperfusion. In addition, an OGD/R PC12 cell model *in vitro* was performed to investigate the expression and role of ATP2B1-AS1 further. As shown in Figure 1C, a similar result was observed that the expression level of ATP2B1-AS1 was increased in the OGD/R PC12 cells compared with the control group and increased in a time-dependent manner for reoxygenation. However, the cell viability of OGD/R PC12 cells was decreased as the reoxygenation time increased (Figure 1D). These results suggested that ATP2B1-AS1 expression was upregulated by cerebral I/R and cellular OGD/R.

### Knockdown of ATP2B1-AS1 Inhibits the Apoptosis in Oxygen and Glucose Deprivation-Reoxygenation PC12 Cells

**FIGURE 2:**
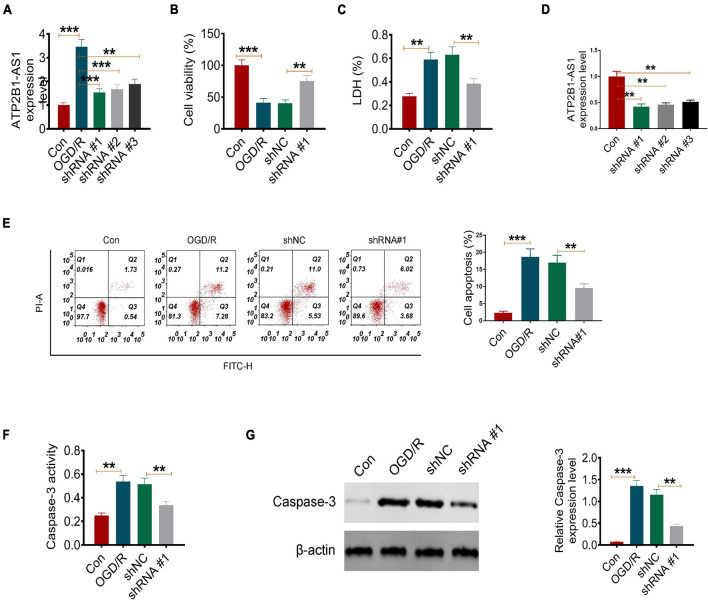
Knockdown of ATP2B1-AS1 inhibits apoptosis and apoptosis-related factors of OGD/R PC12 cells. **(A)** The expression of ATP2B1-AS1 downregulated by shRNA ATP2B1-AS1 in the OGD/R-induced PC12 cells. **(B)** Knockdown of ATP2B1-AS1 increases the cell viability of OGD/R PC12 cells. **(C)** Knockdown of ATP2B1-AS1 reduces the LDH level of OGD/R PC12 cells. **(D)** The expression of ATP2B1-AS1 downregulated by shRNA ATP2B1-AS1 in PC12 cells without OGD/R treatment. **(E)** Knockdown of ATP2B1-AS1 inhibits the apoptosis rate of PC12 cells induced by OGD/R. **(F)** Knockdown of ATP2B1-AS1 decreases the Caspase 3 level of OGD/R PC12 cells. **(G)** Knockdown of ATP2B1-AS1 decreases the Caspase 3 activity of OGD/R PC12 cells. **P < 0.01, ***P < 0.001.

Next, to explore the role of ATP2B1-AS1 in OGD/R-induced PC12 cell apoptosis, vectors carrying shRNA against ATP2B1-AS1 were transfected into the PC12 cells. As shown in Figure 2A, shRNA#1 reduced the ATP2B1-AS1 expression level significantly in the OGD/R-treated PC12 cells, and was used for performing the further experiments. Consistently, the expression of ATP2B1-AS1 was inhibited by shRNA#1 significantly in the PC12 cells without OGD/R treatment (Figure 2D). As shown in Figure 2B, OGD/R induced a low cell viability in the PC12 cells compared with the control group, however, knockdown of ATP2B1-AS1 increased the cell viability compared with the OGD/R and shNC groups significantly. LDH is a cell membrane integrity indicator that indicates cell toxicity. As shown in Figure 2C, the LDH level was increased in the OGD/R group while knockdown of ATP2B1-AS1 decreased OGD/R-evoked LDH release significantly compared to the control group. Moreover, OGD/R induced a high apoptosis rate compared with the control group, however, knockdown of ATP2B1-AS1 suppressed apoptosis compared with the OGD/R and shNC groups significantly (Figure 2E). Caspase-3 is an important apoptosis-associated factor; its activity and expression were also detected in the present study (Figures 2F,G). The results *in vitro* showed that the activity and expression of Caspase-3 were increased remarkably in the OGD/R PC12 cells, but the activity and expression were attenuated by the knockdown of ATP2B1-AS1, indicating that ATP2B1-AS1 may regulate the apoptosis of the OGD/R PC12 cells.

### Knockdown of ATP2B1-AS1 Reduces Middle Cerebral Artery Occlusion/Reperfusion and Oxygen and Glucose Deprivation-Reoxygenation-Induced Inflammation

**FIGURE 3:**
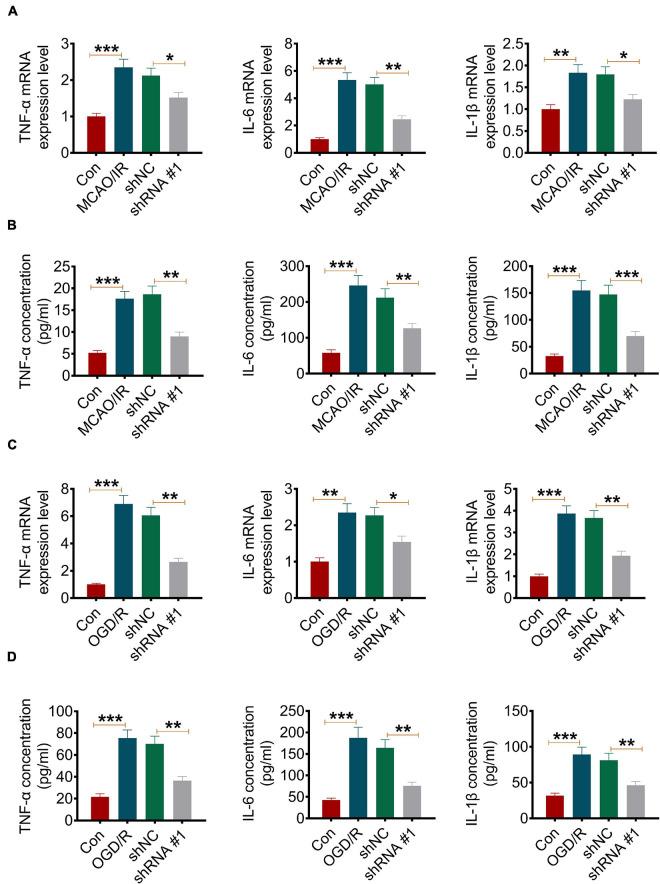
Knockdown of ATP2B1-AS1 decreases the expression of TNF-α, IL-1β, and IL-6 in MCAO/IR rats and OGD/R PC12 cells. **(A)** The mRNA expression of TNF-α, IL-1β, and IL-6 in infarct brain tissues of the MCAO/IR rats was determined by qRT-PCR. **(B)** The content of TNF-α, IL-1β, and IL-6 in infarct brain tissues of MCAO/IR rats was determined by ELISA. **(C)** The mRNA expression of TNF-α, IL-1β, and IL-6 in OGD/R cells was determined by qRT-PCR. **(D)** The content of TNF-α, IL-1β, and IL-6 in OGD/R cells was determined by ELISA. *P < 0.05, **P < 0.01, ***P < 0.001.

It is known that inflammation plays an important role in I/R injury. In the present study, the effect of ATP2B1-AS1 on MCAO/IR and OGD/R-induced inflammation *in vitro* and *in vivo* was evaluated. As shown in Figure 3A, MCAO/IR significantly increased the gene expression, including *TNF-*α, *IL-1*β, and *IL-6*, in the infarct brain tissues compared with the Sham group, while its increasing effect attenuated significantly by the knockdown of ATP2B1-AS1. MCAO/IR also significantly increased the content of the cytokines, including TNF-α, IL-1β, and IL-6, in the infarct brain tissues compared with the Sham group, while its increasing effect attenuated significantly by the knockdown of ATP2B1-AS1 (Figure 3B). In addition, the results of the OGD/R PC12 cells were consistent with the MCAO/IR rat experiment, that shRNA-ATP2B1-AS1 also decreased the mRNA expression and content of these cytokines significantly (Figures 3C,D). Western blot in the PC12 cells also demonstrated that the OGD/R-induced increase in TNF-α, IL-6, and IL-1β expression was recused by the knockdown of ATP2B1-AS1 (Supplementary Figure 1). These results suggested that the knockdown of ATP2B1-AS1 reduced cerebral I/R-induced inflammation.

### ATP2B1-AS1 Functions as a Molecular Sponge of miR-330-5p

**FIGURE 4:**
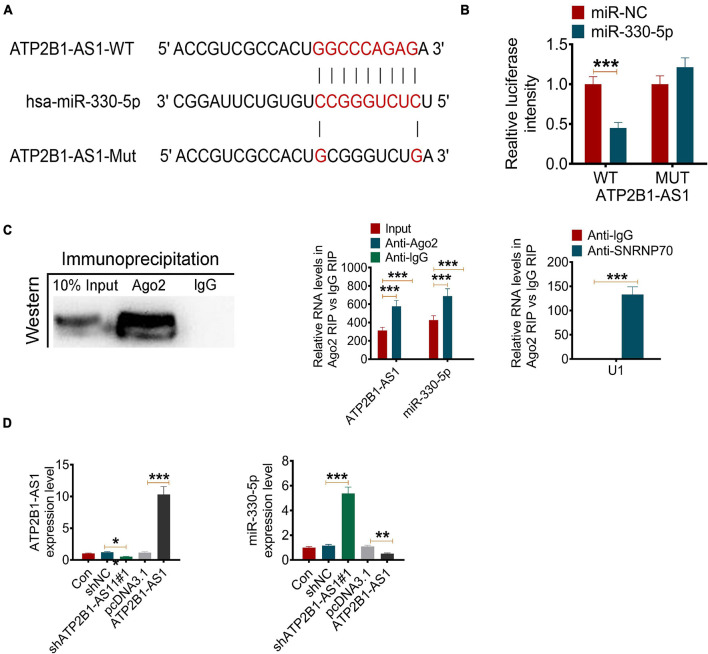
ATP2B1-AS1 targets miR-330-5p. **(A)** The possible interact target of ATP2B1-AS1 was predicted by StarBase. ATP2B1-AS1 could bind miR-330-5p at 3’-UTR. **(B,C)** The binding of ATP2B1-AS1 and miR-330-5p was validated by the dual-luciferase reporter assay **(B)** and RIP assay **(C)**. **(D)** Effect of sh-ATP2B1-AS1 and ATP2B1-AS1 overexpression on the expressions of ATP2B1-AS1 and miR-330-5p. *P < 0.05, **P < 0.01, ***P < 0.001.

A number of studies have revealed that lncRNA plays an important role in post-transcriptional regulation, which can interfere with the miRNA pathway and function as a natural miRNA sponge to reduce the binding of endogenous miRNA and its target genes (Simion et al., 2019). Bioinformatics prediction by StarBase suggested that ATP2B1-AS1 might bind miR-330-5p at 3′-UTR (Figure 4A). To confirm whether ATP2B1-AS1 directly recognizes the 3′-UTR of miR-330-5p, dual-luciferase reporter assay and RIP assay were performed. As shown in Figure 4B, results from the dual-luciferase reporter assay showed that miR-330-5p mimics only reduce the luciferase activity of the wild-type (WT) ATP2B1-AS1 reporter but not that of the Mutant (MUT) one, indicating that miR-330-5p could interact with ATP2B1-AS1. Furthermore, the results of the RIP assay provided further validation for whether ATP2B1-AS1 functions as a sponge (Figure 4C). In addition, our results also suggested that overexpression of ATP2B1-AS1 by transfecting with pcDNA3.1-ATP2B1-AS1 decreased the expression of miR-330-5p while knockdown of ATP2B1-AS1 by transfecting with sh-ATP2B1-AS1 increased the expression of miR-330-5p in the PC12 cells (Figure 4D).

### miR-330-5p Targets TLR4

**FIGURE 5:**
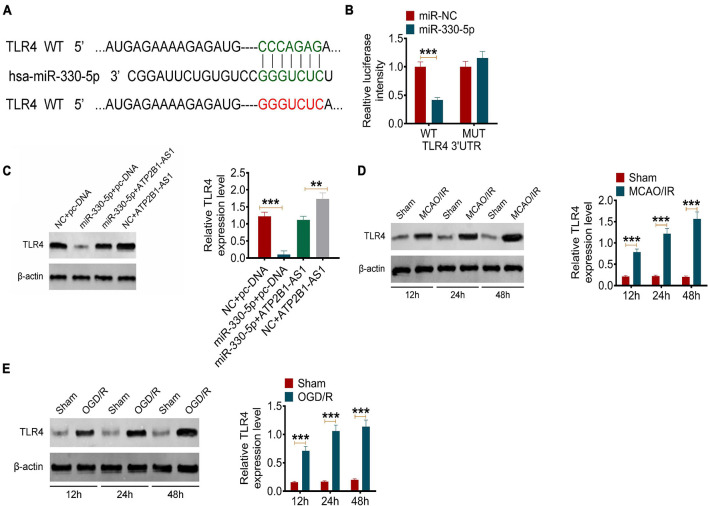
MiR-330-5p targets TLR4 and the expression of TLR4 in MCAO/IR rats and OGD/R PC12 cells. **(A)** The target of miR-330-5p was predicted by TargetScan. MiR-330-5p could bind TLR4 3′UTR. **(B)** The binding of miR-330-5p and TLR4 was validated by a dual-luciferase reporter analysis. **(C)** The effect of ATP2B1-AS1 and miR-330-5p on the expression of TLR. **(D)** The protein expression of TLR4 in MCAO/IR rats for reperfusion for 12, 24, and 48 h was determined by Western blot. **(E)** The protein expression of TLR4 in OGD/R PC12 cells for reoxygenation for 12, 24, and 48 h was determined by Western blot. **P < 0.01, ***P < 0.001.

Next, the target of miR-330-5p was predicted by TargetScan. According to the bioinformatics prediction, miR-330-5p could target TLR4 (Figure 5A). This prediction was further verified by the dual-luciferase reporter assay. The results showed that miR-330-5p mimics only reduce the luciferase activity of the wild-type (WT) TLR4 reporter but not that of the Mutant (MUT) one (Figure 5B). The effect of ATP2B1-AS1 and miR-330-5p on the TLR4 expression was further investigated. Our results showed that ATP2B1-AS1 decreasing inhibited TLR4 expression, and ATP2B1-AS1 increasing promoted TLR4 expression (Supplementary Figure 2). As shown in Figure 5C, the overexpression of miR-330-5p by miR-330-5p mimics decreased the expression of TLR4 and the overexpression of ATP2B1-AS1 by pcDNA3.1-ATP2B1-AS1 increased the expression of TLR4 significantly, however, when miR-330-5p mimics and pcDNA3.1-ATP2B1-AS1 were co-transfected simultaneously, the inhibiting effect of miR-330-5p mimics was abrogated by pcDNA3.1-ATP2B1-AS1. Furthermore, the expression of TLR4 in MCAO/IR rats and OGD/R PC12 cells was further determined. As shown in Figures 5D,E, the expression level of TLR4 was increased in MCAO/IR rats and OGD/R PC12 cells and in a time-dependent manner for reperfusion or reoxygenation. These results suggested that miR-330-5p targets TLR4 and the expression of TLR4 is regulated by ATP2B1-AS1 and miR-330-5p.

### ATP2B1-AS1 Regulates Apoptosis and Inflammatory Response via the miR-330-5p-Mediated TLR4-MyD88-NF-κB Signaling Pathway

**FIGURE 6:**
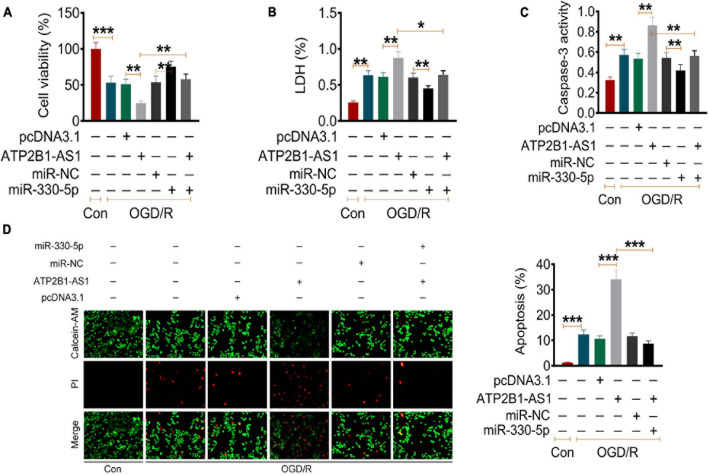
Effect of ATP2B1-AS1 and miR-330-5p on the cell viability, apoptosis, cytokines, and TLR4-MyD88-NF-κB signaling pathway in OGD/R PC12 cells. **(A–D)** The effect of pcDNA3.1-ATP2B1-AS1 and miR-330-5p mimics on the cell viability **(A)**, LDH level **(B)**, Caspase 3 activity **(C)**, and apoptosis rate **(D)** of OGD/R PC12 cells. PcDNA3.1-ATP2B1-AS1 reduced the cell viability, increased the LDH level, Caspase 3 activity, and apoptosis rate of OGD/R PC12 cells. These effects of ATP2B1-AS1 on OGD/R PC12 cells were abrogated by miR-330-5p mimics. *P < 0.05, **P < 0.01, ***P < 0.001.

Our results have demonstrated that ATP2B1-AS1 is highly expressed in MCAO/IR rats and OGD/R PC12 cells and regulates apoptosis and inflammation. Thus, we further explored the relationship of ATP2B1-AS1 and miR-330-5p and their underlying mechanism in the OGD/R PC12 cells. As shown in Figure 6A, our data suggested that OGD/R reduced the cell viability, overexpression of ATP2B1-AS1 further downregulated the cell viability, while the increase of miR-330-5p partly reversed the decrease in cell viability. Importantly, when miR-330-5p mimics was co-transfected simultaneously, the effect of ATP2B1-AS1 on the OGD/R PC12 cells was abrogated. As shown in Figure 6B, our data suggested that OGD/R increased the LDH level, overexpression of ATP2B1-AS1 further increased the LDH level, while the increase of miR-330-5p partly reversed the LDH level. Importantly, when miR-330-5p mimics was co-transfected simultaneously, the effect of ATP2B1-AS1 on the OGD/R PC12 cells was abrogated. Our data also suggested that OGD/R increased the cleaved caspase-3 activity and apoptosis of PC12 cells, overexpression of ATP2B1-AS1 worsened these OGD/R-induced effects, while the increase of miR-330-5p partly reversed these OGD/R-induced effect. Importantly, when miR-330-5p mimics was co-transfected simultaneously, these effects of ATP2B1-AS1 on the OGD/R PC12 cells were abrogated. Furthermore, the effect of ATP2B1-AS1 and miR-330-5p on the OGD/R-induced inflammation in PC12 cells was evaluated (Figures 6C,D).

**FIGURE 7:**
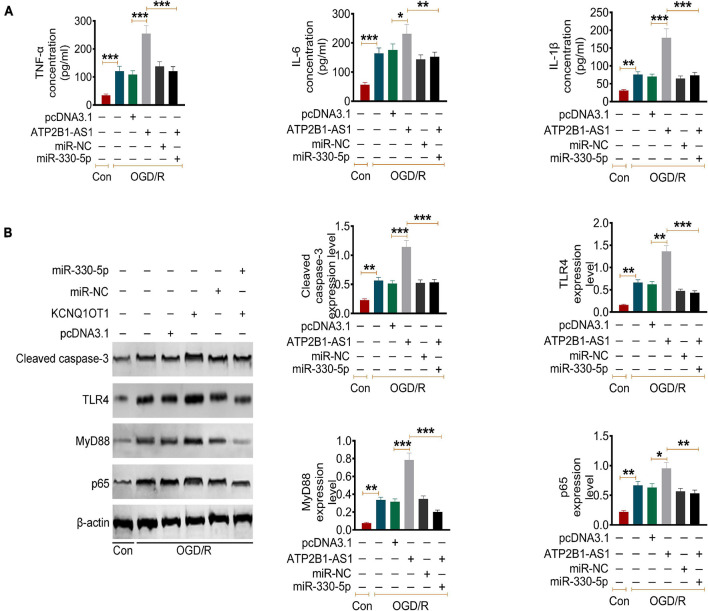
Effect of ATP2B1-AS1 and miR-330-5p on cytokines and the TLR4-MyD88-NF-κB signaling pathway in OGD/R PC12 cells. **(A)** The effect of pcDNA3.1-ATP2B1-AS1 and miR-330-5p mimics on the cytokines levels in OGD/R PC12 cells. PcDNA3.1-ATP2B1-AS1 reduced the level of TNF-α, IL-1β, and IL-6 of OGD/R PC12 cells. These effects of ATP2B1-AS1 on the OGD/R PC12 cells were abrogated by miR-330-5p mimics. **(B)** The effect of pcDNA3.1-ATP2B1-AS1 and miR-330-5p mimics on the expressions of cleaved Caspase 3, TLR4, MyD88, and NF-κB p65 in OGD/R PC12 cells. PcDNA3.1-ATP2B1-AS1 increased the expressions of cleaved Caspase 3, TLR4, MyD88, and NF-κB p65, miR-330-5p mimics abrogated the increasing effect of pcDNA3.1-ATP2B1-AS1. *P < 0.05, **P < 0.01, ***P < 0.001.

Furthermore, the effect of ATP2B1-AS1 and miR-330-5p on the OGD/R-induced inflammation in the PC12 cells was evaluated. Similar results were observed as shown in Figure 7A, OGD/R increased the expression of the level of TNF-α, IL-1β, and IL-6, overexpression of ATP2B1-AS1 further promoted this increases, while this promotion effect of ATP2B1-AS1 was abrogated when miR-330-5p mimics was co-transfected simultaneously. Our previous prediction and validation suggested that miR-330-5p could target and regulate TLR4 expression. Numerous evidence has revealed that the TLR4 and TLR4-mediated MyD88-NF-κB signaling pathway play an important role in the regulation of the inflammatory reaction in a cerebral ischemia injury (Xiaohong et al., 2019; Zhang and Zhang, 2020). Therefore, we hypothesized that ATP2B1-AS1 and miR-330-5p may regulate apoptosis and inflammation via the TLR4-MyD88-NF-κB signaling pathway in a cerebral I/R injury. So, we explored the expression of key proteins in this signaling pathway, as shown in Figure 7B. The results showed that the expression of TLR4, MyD88, and NF-κB p65 was increased after OGD/R, indicating that the TLR4-MyD88-NF-κB signaling pathway was activated. Overexpression of ATP2B1-AS1 enhanced the activation of the TLR4-MyD88-NF-κB signaling pathway; however, this activation was inhibited by the overexpression of miR-330-5p simultaneously.

### Knockdown of ATP2B1-AS1 Improves the Neurological Function of Middle Cerebral Artery Occlusion/Reperfusion Rats

**FIGURE 8:**
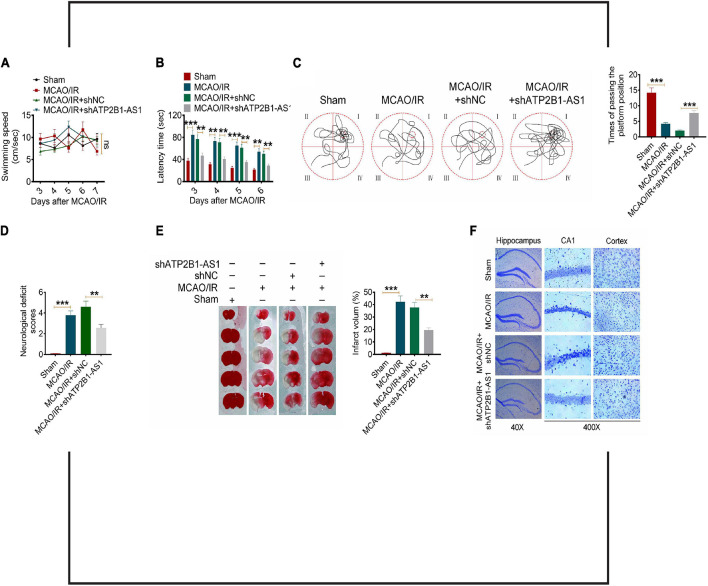
Effect of ATP2B1-AS1 on the neurological function of MCAO/IR rats. A Morris water maze test was performed to evaluate the memory deficits and nerve damage of MCAO/IR rats. **(A)** The swimming speed of all the groups of rats. **(B)** Knockdown of ATP2B1-AS1 decreased the latency time of MCAO/IR rats. **(C)** Knockdown of ATP2B1-AS1 increased the time of passing the platform position of MCAO/IR rats. **(D–F)** Knockdown of ATP2B1-AS1 decreased the neurological deficit scores **(D)**, reduced the cerebral infarct volume **(E)**, and reduced the number of damaged neurons **(F)** of MCAO/IR rats. *P < 0.05, **P < 0.01, ***P < 0.001.

To extend the *in vitro* effect of ATP2B1-AS1 to the *in vivo* situation, we performed a Morris water maze test to evaluate the effect of ATP2B1-AS1 on the neurological function and injury of the MCAO/IR rats. As shown in Figures 8A–C, there was no significant difference for all groups for the swimming speed, but the MCAO/IR and MCAO/IR + shNC groups rats showed a longer latency time and a shorter time of passing the platform position, knockdown of ATP2B1-AS1 decreased the latency time and increased the time of passing the platform position significantly. Furthermore, a high neurological deficit score and serious cerebral infarct were found in the MCAO/R rats, knockdown of ATP2B1-AS1 reduced the cerebral infarct volume and decreased the neurological deficit scores (Figures 8D,E). Knockdown of ATP2B1-AS1 also reduced the number of damaged neurons characterized by shrinkage and nuclear chromatin condensation (Figure 8F). These data suggested that the knockdown of ATP2B1-AS1 significantly improved the cognitive function and nerve damage of the MCAO/IR rats.

## Discussion

A number of studies have reported that lncRNAs are involved in the regulation of cerebral I/R injury. In the present study, we investigated the expression and function of ATP2B1-AS1 in the process of a cerebral I/R injury. The potential neuroprotective effect of ATP2B1-AS1 on the cerebral I/R injury in MCAO/IR rats and OGD/R PC12 cells were explored in our study. As reported in a previous study, ATP2B1-AS1 was highly expressed in mice with myocardial infarction, while silencing of mouse ATP2B1-AS1 reduced cardiomyocyte apoptosis and inhibited the expression of inflammatory cytokines, as well as promoted the cardiomyocyte viability (Song et al., 2020). In this study, we confirmed that the expression of ATP2B1-AS1 was upregulated in a MCAO/IR rat model and OGD/R PC12 cells with a time independent manner. To further determine whether the knockdown of ATP2B1-AS1 could alleviate cerebral I/R injury, we used a shRNA against ATP2B1-AS1 to downregulate ATP2B1-AS1 expression and examined the effect of ATP2B1-AS1 on the cell viability and apoptosis of OGD/R PC12 cells. Our results revealed that the knockdown of ATP2B1-AS1 increased the cell viability and decreased the apoptosis of the OGD/R PC12 cells. There is increasing evidence that inflammation is one of the mechanisms of a cerebral I/R injury (Chai et al., 2020; Zhao et al., 2020). A large number of inflammatory cytokines, such as TNF-α, IL-1, and IL-6, was produced during the process of cerebral I/R injury, these cytokines can trigger excessive tissue inflammation and aggravate the progression of brain injury (Xiaohong et al., 2019; Zhang and Zhang, 2020). To determine whether ATP2B1-AS1 was involved in the regulation of the inflammatory response in a cerebral I/R injury, we detected the expression of cytokines in the brain tissue of MCAO/IR rats and OGD/R PC12 cells. The results revealed that the knockdown of ATP2B1-AS1 decreased the content and expression of TNF-α, IL-1β, and IL-6 *in vivo* and *in vitro*. These results indicated that the knockdown of ATP2B1-AS1 suppressed the cerebral I/R-induced inflammation.

In recent years, the biological function of lncRNA in some serious diseases has attracted a lot of attention (Carpenter, 2016; Taniue and Akimitsu, 2021). MiRNAs are a class of non-coding RNA molecules that consist of 21–23 nucleotides, and the function and role of miRNA in the regulation of various biological processes in human were explored extensively (Abd-Aziz et al., 2020; Li Y. et al., 2020). Previous studies have confirmed that lncRNA may act as an endogenous miRNA sponge to reduce the binding of endogenous miRNA to target genes, and cause a malfunction through various pathological and physiological processes (Sun et al., 2019; Wu J. et al., 2020). In this study, we predicted and validated the possible targeted miRNA of ATP2B1-AS1, and we found that ATP2B1-AS1 targeted miR-330-5p. MiR-330-5p is a highly conserved miRNA that is localized to the human chromosome 19 and mouse chromosome 7 (Feng et al., 2017). The biological functions of miR-330-5p are reported to be related with tumor progression, inflammation response, and oxidative stress (Rambow et al., 2014; Feng et al., 2017; Xiao et al., 2018; Jin et al., 2020). Yu et al. (2021) reported that the overexpression of miR-330-5p effectively reduced LPS-induced pulmonary vascular endothelial cell injury, and inhibited expressions of TLR4, NF-κB, and inflammatory cytokines. Xing et al. (2020) investigated that miR-330-5p overexpression inhibited inflammation and oxidative stress by suppressing the TRAF6/NF-κB signaling in LPS-induced septic cardiomyopathy. Furthermore, inhibition of miR-330-5p could increase the protein expressions of NLRP3 inflammasome-related proteins, and miR-330-5p/TIM3 axis regulated the NLRP3 inflammasome-mediated myocardial inflammation (Zuo et al., 2020). However, the role of miR-330-5p in cerebral I/R injury remains unknown. Our results suggested that the overexpression of ATP2B1-AS1 reduced cell viability, promoted apoptosis, and increased the expression of inflammation cytokines (TNF-α, IL-1β, and IL-6) in the OGD/R PC12 cells, however, these effects were abrogated by miR-330-5p.

Our further prediction and validation showed that TLR4, an important membrane receptor that mediates innate immunity, was the targeted mRNA of miR-330-5p. Our results showed that TLR4 expression was upregulated in the infarct area of MCAO/IR rats and in OGD/R PC12 cells. Our results are consistent with the findings from previous studies (Li X. et al., 2020; Yang et al., 2020). When TLR4 is activated, it can regulate the expression of various inflammatory mediators and cytokines through transmembrane signaling, and initiate defense and immune regulation (Rahimifard et al., 2017; Rayees et al., 2020). Studies have shown that TLR4-mediated inflammatory response plays an important role in heart, lung, liver, and cerebral I/R injury (Rayees et al., 2020). After cerebral ischemia, TLR4 recognizes the endogenous substances released from brain tissue such as heat shock protein, fibrinogen, hyaluronic acid, heparan sulfate, etc., and finally activates NF-κB. NF-κB is an important nuclear transcription factor that is involved in many physiological and pathological processes, such as inflammation and apoptosis (Zhou et al., 2020). Within the TLR4 signaling pathway, the MyD88-dependent signaling pathway is an important activator of NF-κB and the subsequent regulatory effects of NF-κB signaling (Zhang et al., 2019). Furthermore, the role of TLR4/MyD88/NF-κB signaling pathway-mediated inflammation in cerebral I/R injury has been confirmed by several studies (Li J. et al., 2019; Li W. et al., 2019; Yan et al., 2019; Gao et al., 2020). Therefore, we hypothesized that the regulation of ATP2B1-AS1 and miR-330-5p in cerebral I/R injury may be achieved through the TLR4-MyD88-NF-κB signaling pathway. The results suggested that the overexpression of ATP2B1-AS1 enhanced the increased expressions of TLR4, MyD88, and NF-κB p65 in the OGD/R PC12 cells, indicating that the TLR4-MyD88-NF-κB signaling pathway was activated, while this activation was inhibited by miR-330-5p. Finally, we examined the effect of ATP2B1-AS1 knockdown *in vivo* by a Morris water maze test. The results showed that the knockdown of ATP2B1-AS1 significantly decreased the latency time, increased the time of passing the platform position, reduced the cerebral infarct volume, decreased the neurological deficit scores, and reduced the number of damaged neurons of the MCAO/IR rats. These data indicated that knockdown of ATP2B1-AS1 improved the cognitive function and nerve damage of MCAO/IR rats remarkably.

## Conclusion

In conclusion, this study first demonstrates that ATP2B1-AS1 is highly expressed in the brain tissue and cells suffered from cerebral I/R injury. ATP2B1-AS1 promotes cerebral I/R injury as a competing endogenous RNA for sponging miR-330-5p through activating TLR4-MyD88-NF-κB signaling pathway. Knockdown of ATP2B1-AS1 has been shown to promote the proliferation and inhibit the apoptosis and inflammation of the OGD/R PC12 cells, improve the cognitive function, and alleviate nerve damage of the MCAO/IR rats. These findings raise the possibility that ATP2B1-AS1 may be an attractive therapeutic target for the treatment of cerebral ischemic injuries. However, there exist some limitations in our study. For instance, TLR4-MyD88-NF-kB and TLR4-TRIF-IRF3 are two critical TLR4-related pathways, while we only focused on the former. The role of the TLR4-TRIF-IRF3 pathway in the regulation of ATP2B1-AS1 to I/R injury remains unclear (Kuzmich et al., 2017). The underlying mechanisms for the ATP2B1-AS1 detrimental effect and some critical proteins or genes involved in the TLR4-MyD88-NF-κB signaling pathway are still needed to be elucidated by further study.

## Data Availability Statement

The original contributions presented in the study are included in the article/Supplementary Material, further inquiries can be directed to the corresponding author/s.

## Ethics Statement

The animal study was reviewed and approved by the Institutional Animal Care and Use Committee of Histology and Embryology, and Institute of Neurobiology, Xi’an Jiaotong University Health Science Center.

## Author Contributions

All authors completed the design and implementation of experiments, data analysis, and manuscript drafting in the union, contributed to the article, and approved the submitted version.

## Conflict of Interest

The authors declare that the research was conducted in the absence of any commercial or financial relationships that could be construed as a potential conflict of interest.

## Publisher’s Note

All claims expressed in this article are solely those of the authors and do not necessarily represent those of their affiliated organizations, or those of the publisher, the editors and the reviewers. Any product that may be evaluated in this article, or claim that may be made by its manufacturer, is not guaranteed or endorsed by the publisher.

## Supplementary Material

Supplementary Figure 1 Knockdown of ATP2B1-AS1 decreases the expression of TNF-α, IL-1β, and IL-6 in OGD/R PC12 cells. The protein levels of TNF-α, IL-1β, and IL-6 in the OGD/R-induced PC12 cells was determined by Western blot. **P* < 0.05, ***P* < 0.01, ****P* < 0.001.

Supplementary Figure 2 Effect of ATP2B1-AS1 on TLR4 expression. The expression of TLR4 in PC12 transfected with shRNA or ATP2B1-AS1 overexpression plasmid was determined by Western blot. ***P* < 0.01.
