# Supplementary figures and images for: ATP2B1-AS1 Promotes Cerebral Ischemia/Reperfusion Injury Through Regulating the miR-330-5p/TLR4-MyD88-NF-κB Signaling Pathway
Source: Front Cell Dev Biol. 2021 Oct 12;9:720468. doi: 10.3389/fcell.2021.720468 (PMC8545896; doi:10.3389/fcell.2021.720468)

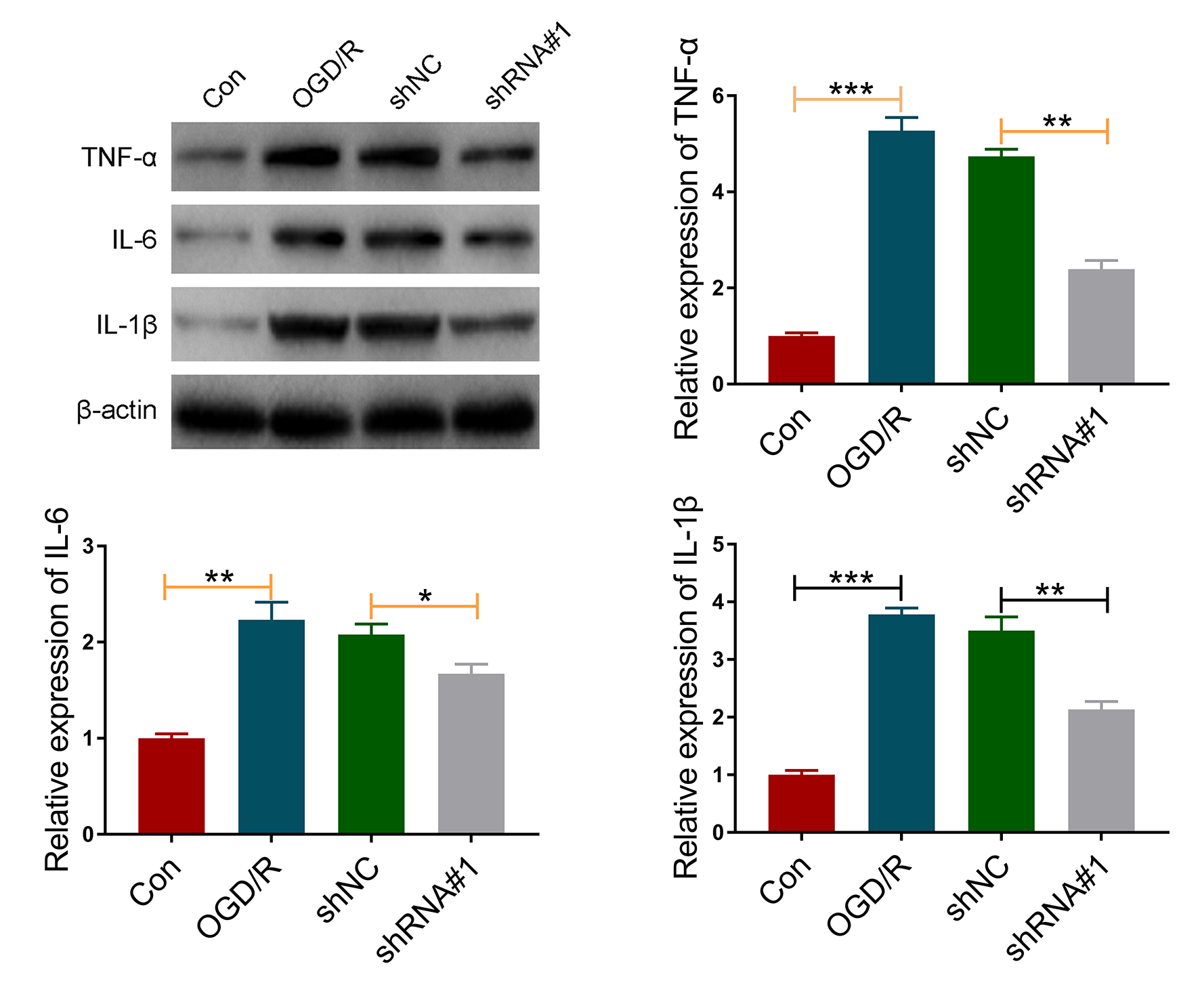

Supplement: Supplementary Figure 1 — Knockdown of ATP2B1-AS1 decreases the expression of TNF-α, IL-1β, and IL-6 in OGD/R PC12 cells. The protein levels of TNF-α, IL-1β, and IL-6 in the OGD/R-induced PC12 cells was determined by Western blot. *P < 0.05, **P < 0.01, ***P < 0.001. [file Image_1.TIF]

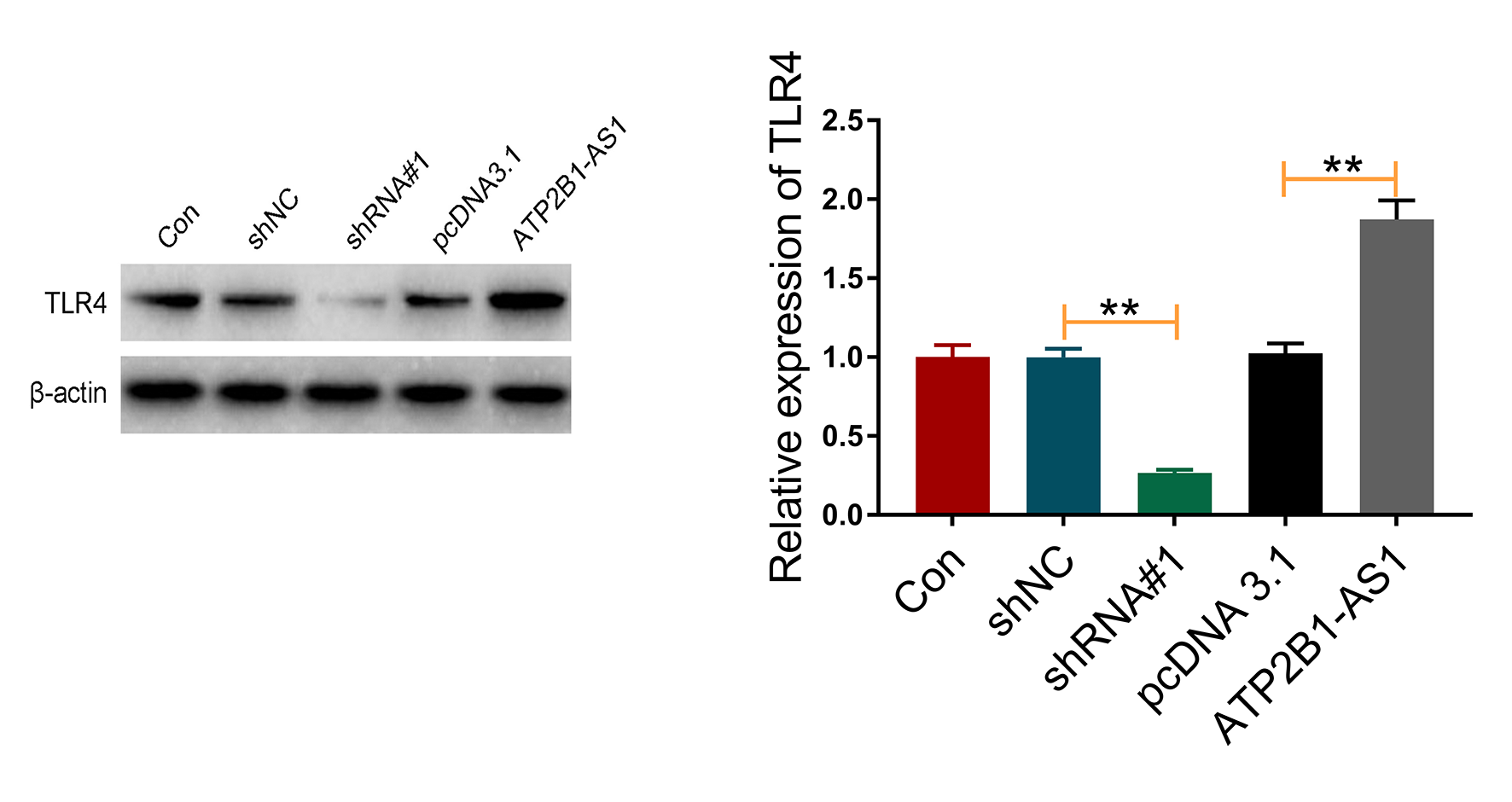

Supplement: Supplementary Figure 2 — Effect of ATP2B1-AS1 on TLR4 expression. The expression of TLR4 in PC12 transfected with shRNA or ATP2B1-AS1 overexpression plasmid was determined by Western blot. **P < 0.01. [file Image_2.TIF]
